# Storylines of family medicine IV: perspectives on practice—lenses of appreciation

**DOI:** 10.1136/fmch-2024-002791

**Published:** 2024-04-12

**Authors:** William B Ventres, Leslie A Stone, Radeeb Akhtar, Jeffrey M Ring, Lucy M Candib, Erick Messias, Ronald M Epstein, Marc Tunzi, Amy L Lee, Christopher P Morley, Carina M Brown, David Slawson, Jill Konkin, David G Campbell, Ian Couper, Susan Williams, Robert Brooks, Lucie Walters

**Affiliations:** 1 Family and Preventive Medicine, University of Arkansas for Medical Sciences College of Medicine, Little Rock, Arkansas, USA; 2 Family Medicine and Community Health, Icahn School of Medicine at Mount Sinai, New York, New York, USA; 3 Independent Health Psychologist, Los Angeles, California, USA; 4 Family Medicine and Community Health, UMass Chan Medical School, Worcester, Massachusetts, USA; 5 Psychiatry and Behavioral Neurosciences, Saint Louis University School of Medicine, Saint Louis, Missouri, USA; 6 Family Medicine, University of Rochester Medical Center, Rochester, New York, USA; 7 Family Medicine Residency Program, Natividad Medical Center, Salinas, California, USA; 8 Family Medicine, Tufts University School of Medicine, Boston, Massachusetts, USA; 9 Public Health and Preventive Medicine, SUNY Upstate Medical University, Syracuse, New York, USA; 10 Cone Health Family Medicine Residency, The University of North Carolina School of Medicine, Greensboro, North Carolina, USA; 11 Family Medicine, Atrium Health, Charlotte, North Carolina, USA; 12 Family Medicine, University of Alberta Faculty of Medicine & Dentistry, Edmonton, Alberta, Canada; 13 Cunninghame Arm Medical Centre, Australian College of Rural and Remote Medicine, Lakes Entrance, Queensland, Australia; 14 Ukwanda Centre for Rural Health, Stellenbosch University Faculty of Medicine and Health Sciences, Cape Town, Western Cape, South Africa; 15 Adelaide Rural Clinical School, The University of Adelaide, Adelaide, South Australia, Australia; 16 Department of Rural Health, Broken Hill University, Broken Hill, New South Wales, Australia; 17 Adelaide Rural Clinical School, The University of Adelaide Faculty of Health and Medical Sciences, Mount Gambier, South Australia, Australia

**Keywords:** Family Medicine, General Practice, Attitude to Health, Community Medicine, Health Knowledge, Attitudes, Practice

## Abstract

*Storylines of Family Medicine* is a 12-part series of thematically linked mini-essays with accompanying illustrations that explore the many dimensions of family medicine, as interpreted by individual family physicians and medical educators in the USA and elsewhere around the world. In ‘IV: perspectives on practice—lenses of appreciation’, authors address the following themes: ‘Relational connections in the doctor–patient partnership’, ‘Feminism and family medicine’, ‘Positive family medicine’, ‘Mindful practice’, ‘The new, old ethics of family medicine’, ‘Public health, prevention and populations’, ‘Information mastery in family medicine’ and ‘Clinical courage.’ May readers nurture their curiosity through these essays.

## Introduction

Physicians are commonly trained to be ‘objective’ in their thinking and ‘clinically detached’ in their professional stance vis-à-vis patients. However, regardless of the medical discipline practised, all physicians bring something of their perspectives on life and the goals of medicine to their interactions with patients. Although this reality is often discounted in traditional medical education, family medicine has consistently aspired to incorporate interpersonal aspects of care into the training of its learners and the work of its practitioners. In the storylines of family medicine essays that follow, the authors explore the points of view that have helped them better appreciate, assess and address their patients’ concerns. We hope they may help you, too, in your work with patients.

## Relational connections in the doctor–patient partnership

Radeeb Akhtar and Jeff Ring

*Medical professionals must pull back the curtain of blindness, neglect and defensiveness and explore the biases, assumptions and actions that lead to discrimination in communication and healthcare decision-making.*

The doctor–patient healing partnership is a relational process built on a sacred footing of respectful engagement woven together with compassionate patient-centred care. The importance of this relational process grows when working across boundaries of culture, especially with minority patients, many of whom often shoulder the physical and mental burdens of health inequities and injustice along with historically rooted barriers to trust. Wise family physicians working across cultural boundaries recognise and ‘bring into check the power imbalances that exist in the dynamics of physician–patient communication by using patient-focused interviewing and care.’^1^

How does one best learn this process? Begin by looking within. It ‘is imperative that there be a simultaneous process of self-reflection (realistic and ongoing self-appraisal) and commitment to a lifelong learning process.’^1^ Be ‘flexible and humble enough to let go of the false sense of security that stereotyping brings.’^1^ Explore one’s own multidimensional cultural background, biases and stereotypes, all the while recognising and respecting the cultural priorities and practices of others. Developing an internal sense of humility—one that is thoughtful, realistic and honest—is the starting point for building partnerships with others.

Curiosity is a characteristic well worth cultivating across one’s professional career, particularly in the face of uncertainty and discomfort in challenging clinical encounters.^2^ Lean into curiosity when considering how to navigate power, develop cultural and racial humility, understand implicit biases and reflect on one’s own history. Intentional engagement becomes an outward investment towards building trust with patients and others, including family members and community. Tactful verbal and non-verbal communication can transform routine patient care into healing rituals. Incorporate active listening, remain present while working with patients and when needed, seamlessly integrate interpreters into the visit. Dip deeply into the well of appreciation.

Of paramount importance is self-reflection. The psychiatrist Roberto Montenegro writes about humility, bias, the pain of invisibility and the sting of discrimination in his essay, ‘My Name is Not ”Interpreter”.’^3^ He retells personal experiences of having been on both sides—the delivering as well as the receiving ends of cultural blindness, incorrect assumptions and hurtful communication. His story exemplifies a depth of open and non-defensive self-reflection that is courageous, vulnerable and steeped in cultural humility. Learning from experience helps build and refine one’s humanity, the demonstration of which is so important in interactions with patients.

Family physicians must grow their capacities for deep listening to others and to themselves. This is particularly important when serving underserved patients, many of whom have experienced marginalisation because of systemic racism and other adverse social determinants. The quality of the healthcare these patients receive depends on the degree to which individual physicians work to listen and understand.

**Figure 1:**
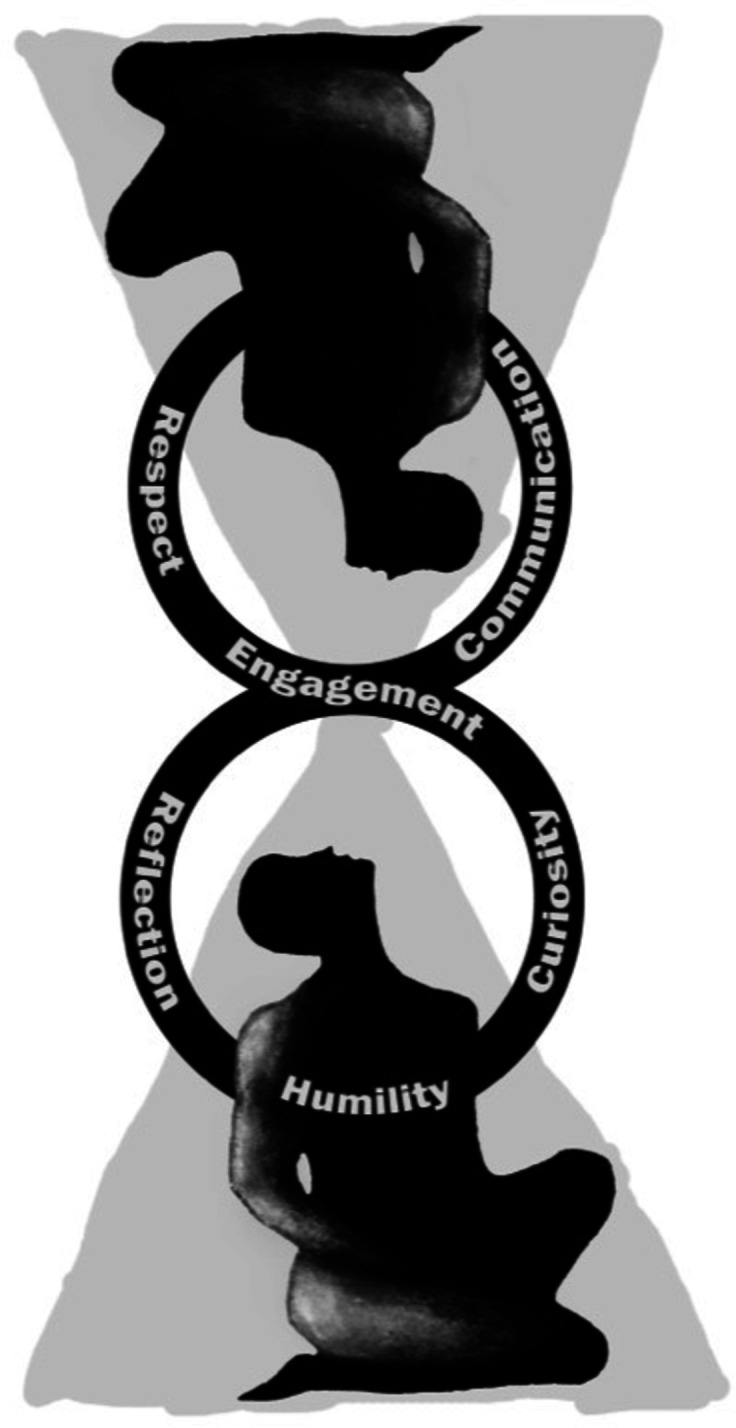
Exploring culture through a compassionate gaze. Reproduced with permission.^5^

Whether in a clinic, hospital or community-based setting, cultural humility, curiosity, respectful interpersonal engagement and empathic understanding form the foundation of optimal relationship-centred care.^4^ May family physicians everywhere cultivate these characteristics and become potent advocates for the improved delivery of healthcare and the upstream prevention of racism and discrimination (figure 1).^5^

### Readings

- Dyche L, Epstein RM. Curiosity and medical education. *Med Educ* 2011;45:663–8. doi: 10.1111/j.1365–2923.2011.03944 .x
- Montenegro RE. My name is not ‘interpreter’. *JAMA* 2016;315:2071–2. doi: 10.1001/jama.2016.1249
- Tervalon M, Murray-Garcia J. Cultural humility vs cultural competence: a critical distinction in defining physician training outcomes in multicultural education. *J Health Care Poor Underserved* 1998;9:117–25. doi: 10.1353/hpu.2010.0233

## Feminism and family medicine

Lucy Candib

*Feminism and family medicine are entwined because gender and power are integral to how individuals experience their bodies, their own health and illness concerns, their understandings of sickness among family members and friends, and their interactions with the healthcare system.*

When I speak of feminism and family medicine, I explore how clinicians view healthcare.^6^ Do they and their coworkers consider the family and the relational context of their patients? Do they consider how their gender affects the patients before them and vice versa? Are they aware of the power differentials that arise from differences in education, technical expertise and income (and often race, nationality and heteronormative status as well)?

Feminism helps us address these issues—families, relationships, gender and power. All are central to understanding not only the concepts of sickness and health within families and communities but also the process of healthcare and the intention to cure and heal.

Years ago, as a budding feminist, I chose family medicine as a way to work as an advocate for women within a system that pathologised women’s health. During my rotating internship at a public hospital, I loved taking care of children; I did not, however, like how their mothers were treated. If the mother brought in a kid who was not particularly sick, why did they bother bringing the child in? If they brought in a kid who was very sick, why did they wait so long to visit the doctor? In a child-focused setting, I could not find a way to recognise and support the hard work of women amid their overwhelming, and often resource-limited, worlds.

I needed to work with women in a way that transcended their parental role, especially at a time when women’s healthcare was limited to the then male-dominated, primarily surgical specialty of obstetrics and gynaecology. I wanted to provide reproductive care that empowered women to make healthy choices that would work for them. I also saw the need to help individuals and couples transcend harmful gender roles that had been learnt from prior generations and instead learn respectful ways of relating to each other and their children.

With this vision, I worked to uncover how physical violence and sexual violence—often unrecognised and usually unmentioned—influence how girls and women view themselves and limit their ability to be strong and healthy.^7^ (Of course, comparable forces shape boys and men. Although not my primary focus, these forces deserve attention also: gendered expectations constrict the health and safety of everyone.) I realised that girls and adolescent women need to make decisions based on their personal needs and desires; I came to understand the concerns of women in their middle years, as they face distinctive social, physical and structural limitations, particularly in a society where gendered expectations inhibit people’s abilities to build safe spaces for themselves and their loved ones to grow.^8^

Family physicians aspire to make things right for patients and their families in an economic and social world designed to maintain power imbalances that underlie racial, class, ethnic, gender and sexual vulnerabilities.^9^ Family physicians strive to empower all and help patients to grow as individuals and as members of families and communities.

**Figure 2:**
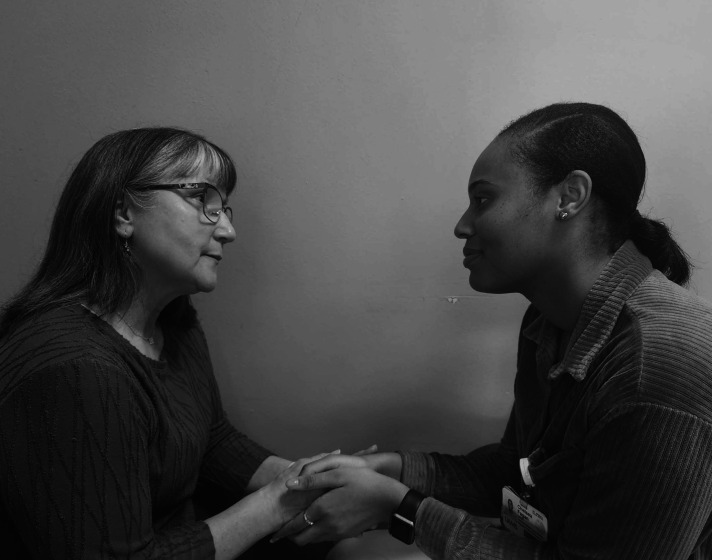
Feminism embodied. Reproduced with permission.^10^

These are huge tasks to be sure. So where to start? Consider this core tenet borrowed from feminist thought: Share the power entrusted in us with those we hope to help towards health and healing (figure 2).^10^

### Readings

- Candib LM. Ways of knowing in family medicine: contributions from a feminist perspective. *Fam Med* 1988;20:133–6.
- Candib LM. Working with suffering. *Patient Educ Couns* 2002;48:43–50. doi:10.1016/s0738-3991(02)00098–8
- Candib LM. Sí, doctora. *Ann Fam Med* 2006;4:460–2. doi:10.1370/afm.572

## Positive family medicine

Bill Ventres and Erick Messias

*Using a positive family medicine approach, family physicians can apply principles and tools borrowed from positive psychology to treat patients in need.*

Because of the relational nature of family medicine, family physicians have a duty to hone their abilities to engage with patients therapeutically. Many have categorised these skills in terms of effective communication with patients^11^; some have even formulated checklists for evaluating communicative behaviours during clinical encounters.^12^

However, communicative proficiency in family medicine requires more than just attending to interpersonal conduct in the exam room. Proficiency in the intersubjective practice of family medicine—the relational mix in which doctors and patients find themselves when they come together—requires deep insight into the nature and use of healing bonds as both inspirational guides and therapeutic instruments.^13^

To build this kind of proficiency, we suggest that learners of family medicine—and we all are learners—borrow principles from the discipline of positive psychology and work to integrate these principles into their care of patients.^14^

Like existential psychology, positive psychology incorporates the study of positive emotions into psychology and psychiatry’s traditional focus on negative emotions and psychopathology. In addition, positive psychology has introduced new approaches to clinical practice. Many of these approaches have been empirically validated, enabling practising psychotherapists and psychiatrists to supplement their therapeutic toolboxes with positive interventions.

**Figure 3:**
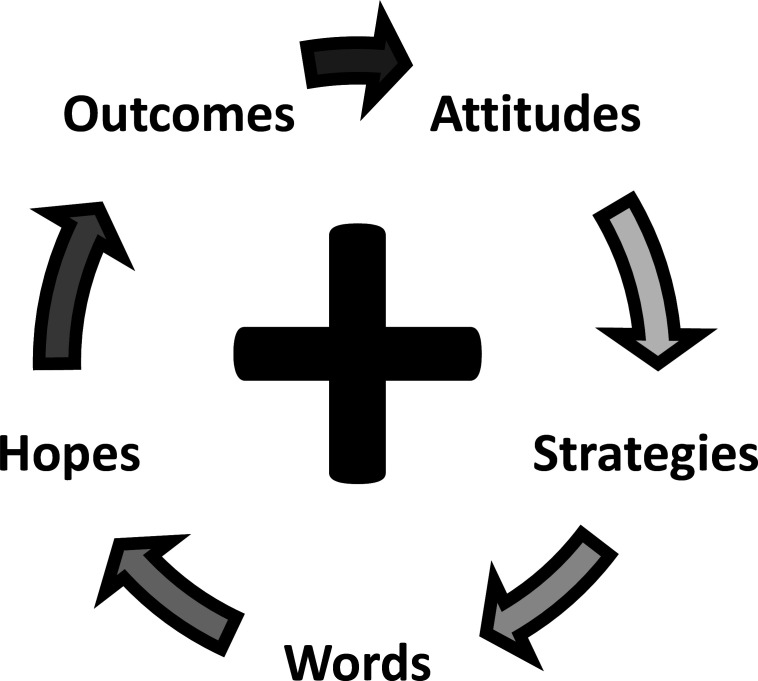
Putting positive family medicine into practice: key features.

Of course, family medicine is not limited to patients with mental health concerns, and we, therefore, suggest incorporating positive psychology into positive family medicine by integrating five positive principles into daily practice (figure 3):

- **Hold positive attitudes**—See patients, despite their limitations, as worthy of esteem and able to endure illness with dignity and grace.
- **Employ positive strategies**—Involve patients as equal partners in addressing their concerns and frame recommendations as investments in the future.
- **Speak positive words**—Use keywords and phrases that inspire patients to create their own visions of a healthier future, regardless of past medical or social histories.
- **Express positive hopes**—Encourage goals that explicitly affirm patients’ personal strengths and virtues.
- **Envision positive outcomes**—Guide patients to aim high while nudging them towards healthier behaviours, despite the challenging realities of life that they may endure.

Importantly, practising positive family medicine means shifting our overall clinical stance vis-à-vis patients from a traditional disease-oriented, deficit-focused point of view to one that emphasises building personal resiliency and collective capacity in the face of illness.^15^

Practising positive family medicine does not mean ‘prescribing’ happiness.^16^ Instead, it means consciously considering patients’ potential for realising progress in moving towards health. It summons us to lead patients towards improved clinical outcomes from a space of appreciation and optimism. It invites us to engage patients with words that encourage and approaches that foster collaboration and commitment.

### Readings

- Friedman SE, Levy E. Applying positive psychology to the practice of medicine. *MGMA Connection* 2014;14:28–31.
- Hershberger PJ. Prescribing happiness: positive psychology and family medicine. *Fam Med* 2005;37:630–4.
- Ventres WB. How I think: perspectives on process, people, politics, and presence. *J Am Board Fam Med* 2012;25:930–6. doi: 10.3122/jabfm.2012.06.120093

## Mindful practice

Ron Epstein

*Mindful practice refers to a purposeful attentiveness to one’s inner life—thoughts, bodily sensations and emotions—during everyday work, with the goal of greater clarity, insight, effectiveness, wisdom and compassion.*

The secularised concept of mindful practice finds its source in Buddhist virtue ethics that emphasises the connection between one’s quality of mind and promoting good in the world. The word ‘mindfulness,’ in Pali, connotes ‘remembering’ and is usually characterised as ‘right mindfulness.’ Right mindfulness suggests that, through practice, we can more skillfully enact ‘the most important thing’ in each moment,^17^ even in the chaos and uncertainties of clinical practice.^18^

Four qualities of mindful practitioners are particularly relevant in clinical medicine.^19^ First is attentive observation of oneself and the environment, recognising that ‘we don’t see things as they are; we see things as we are.’^20^ Knowing the lens through which we view the world (and ourselves) opens up the possibility of recognising our biases and triggers, taking a pause and recalibrating our perceptions so that we may see things as they are rather than how we would have them be.

Second, critical curiosity is the capacity to remain open and actively seek new information and experiences, taking interest in the person with the illness and not just the diagnostic imperative.^20 21^ Remaining curious is particularly important—and challenging—for clinicians when they feel stressed, and when they face uncertainty, ambiguity or unforeseen negative experiences.

Third, beginner’s mind refers to the capacity to see novelty in the familiar, to see a situation from more than one perspective while avoiding premature closure. Recognising that ‘in the beginner’s mind the possibilities are many, in the expert’s mind they are few,’^17^ adopting a beginner’s mind is particularly important when we feel stuck, when our actions are not producing desired results and when we need to think outside the box of our current understanding.

Fourth, presence refers to a quality of ‘being there’, the exhilaration of being immersed in a task, and—of critical importance when practising medicine—a sense of shared mind and authentic feeling with patients, families and colleagues that ‘occurs in the space between them as much as in their emotions.’^22^

Mindful practice involves a moral stance. Mindfulness is a vector, pointing to values and attributes that are foundational to clinical practice. Attitudes of mind—attentive observation, critical curiosity, beginner’s mind and presence—are cultivated with the purpose of promoting prosocial goals: listening to understand and not merely respond, enabling and empowering patients towards health, being technically adept, recognising and responding to our own errors, communicating effectively in emotionally charged and conflict-ridden situations, engaging in compassionate actions even when they are not required by our professional codes, and self-care in order to sustain ourselves and be available to those who need us the most.

**Figure 4:**
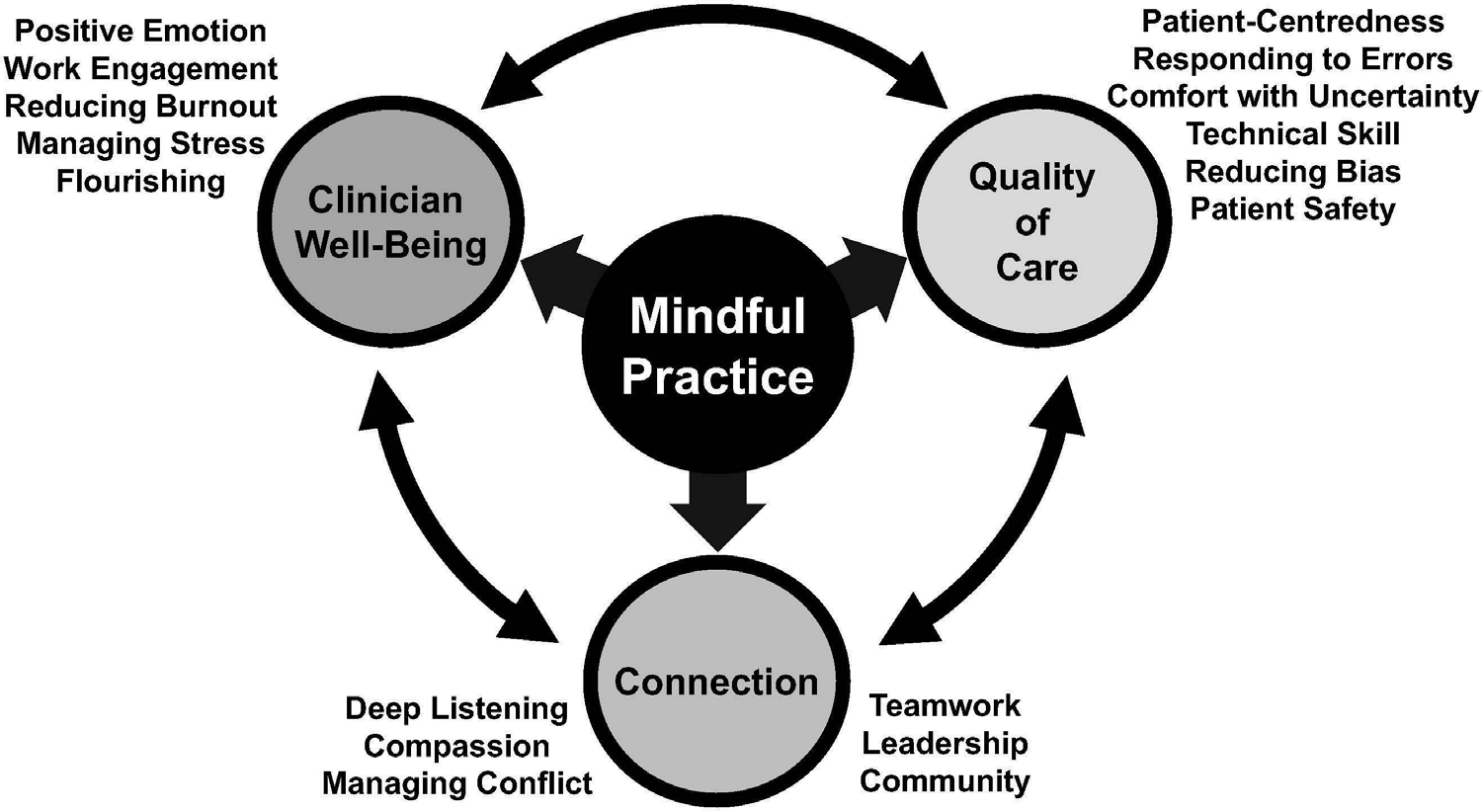
Foundations of mindfulness in medicine.

Mindful practice can be taught and cultivated. Training in mindful practice involves contemplative practices, sharing and listening deeply to narratives about meaningful and difficult moments, and taking an appreciative, strengths-based approach. Contemplative practices can be brought to the workplace—for example, we encourage clinicians to prepare for a patient visit by pausing briefly; taking a breath; being aware of thoughts, feelings and bodily sensations in the moment; and discerning which feelings, attitudes and skills to bring with them into the room and which to temporarily set aside. These and other practices help to develop shared presence, clear communication and a sense of community among clinicians and also to help mitigate burnout, moral distress and isolation (figure 4).^23–25^

### Readings

- Borrell Carrió F, Epstein RM. Preventing errors in clinical practice: a call for self-awareness. *Ann Fam Med* 2004;2:310–6. doi: 10.1370/afm.80
- Epstein RM. Mindful practice. *JAMA* 1999;282:833–9. doi: 10.1001/jama.282.9.833
- Fitzgerald FT. Curiosity. *Ann Int Med* 1999;130:70–2. doi: 10.7326/0003-4819-130-1-199901050-00015

## The new, old ethics of family medicine

Bill Ventres and Marc Tunzi

*Family physicians see medical ethics as more than just solving problems; it’s a way of being in relationship with patients.*

There are many lenses by which to understand how to practise medicine ethically. These include casuistry, the ethics of care, feminist ethics, narrative ethics, virtue ethics and microethical decision-making.^26^ Nonetheless, most clinicians continue to use the four principles as guides to discuss key ethical topics in medicine and manage ethical conflicts as they arise. These four principles include^27^:

- **Autonomy**—Individuals have the right to control their own bodies and make their own decisions (barring negative consequences to others).
- **Non-maleficence**—Do no harm.
- **Beneficence**—Do good.
- **Justice**—Treat everyone equitably.

Although these four principles can be quite useful, especially when attending to specific problems needing ethical consideration, they often do not fit the context of family medicine. The aim of family medicine is to provide care that is simultaneously relationship-based, community-focused, patient-centred, accessible, comprehensive, continuous, coordinated and contextual.^26^ Thus, ethical considerations made in family medicine are different than those made in most other medical fields. Ethics in family medicine is an ethos of practice that implies a way of being with patients, routinely and over time. Remember, the word ethics comes from the Greek word *ethos*, which means custom or habit.

This ethos of practice honours five ‘T’ habits of mind and action^28^:

- **Employ *time* wisely**—Know that many decisions in family medicine don’t have to be made in the moment, and planning can lessen the probability of future discord.
- **Engage patients and families with the give-and-take of *talk***—Communicating critical points of view can help with mutual understanding.
- **Exercise *tact***—Make the ethical choice to see and recognise patients as people worthy of dignity and respect.
- **Employ *touch* therapeutically**—Demonstrate empathy through appropriate and caring physical examination skills.
- **Engender *trust***—Develop trust over time, bit by bit, in the day-to-day work of attending not only to patients’ concerns but also to all that lies behind the veil of those concerns, the experience of illness in all its dimensions.

One of the founders of academic family medicine developed four principles specific to his care of patients. These principles exemplify how the ethics of family medicine revolves around relationship and how family physicians conceptualise their work. These principles are as important now as they were when first described some 40 years ago^29^:

- **Affinity**—The special, therapeutic bond between physician and patient.
- **Intimacy**—The openness that results from the existence of affinity.
- **Reciprocity**—The mutual, bidirectional sense of trust that emerges from intimacy.
- **Fidelity**—The expectation of being there, for and with the patient, in the future.

**Figure 5:**
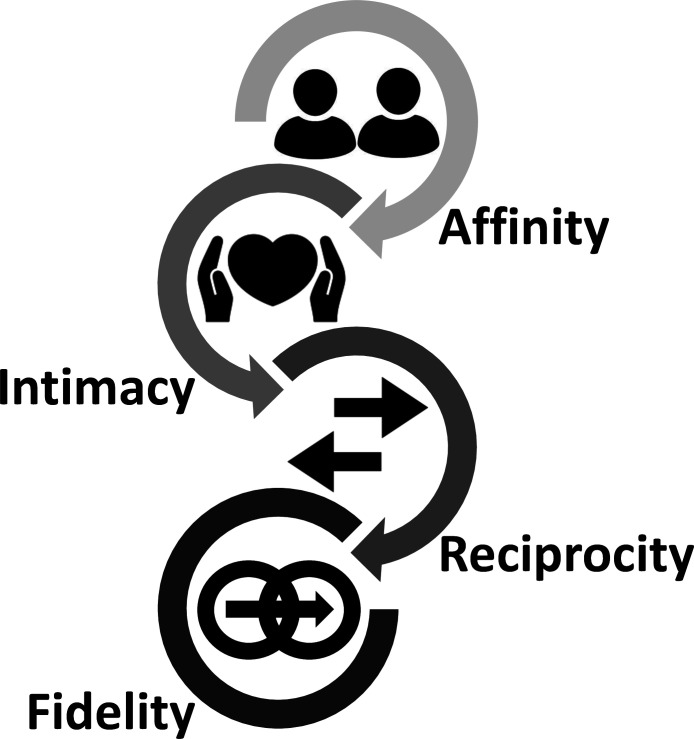
Family medicine’s four principles.

These four principles characterise a functioning therapeutic relationship. Collectively, they define the process of care that describes family medicine ethics and defines family medicine (figure 5).

### Readings

- Carmichael L. Voices from family medicine: Lynn Carmichael. Interview by William B. Ventres and John J. Frey. F*am Med* 1992;24:53–7.
- Tunzi M, Ventres W. Family medicine ethics: an integrative approach. *Fam Med* 2018;50:583–8. doi: 10.22454/FamMed.2018.821666
- Ventres W, Tunzi M. Ways of being in generalist practice: using 5 ‘T’ habits of mind to guide ethical behavior. *J Clin Ethics* 2020;31:184–90. doi: 10.1086/JCE2020312184

## Public health, prevention and populations

Amy Lee and Chris Morley

*Many public health concepts are essential to medical education and clinical care. Family physicians are well positioned to use public health approaches to improve the health of individuals of all ages, family units, communities and populations.*

Public health encompasses laws, policies, institutional and agency regulations, and many other aspects of society that collectively ‘assure the conditions in which people can be healthy.’^30^ Public health includes traditional disciplines of epidemiology and biostatistics, programme planning, law and policy, health behaviour, environmental health, risk assessment and a host of other subspecialties.

Population health concerns health outcomes within a distinct group of individuals, such as a clinical panel or the catchment area of a healthcare organisation and the distribution of outcomes across subgroups within those populations.^31 32^ The concept of population health recognises ‘an opportunity for healthcare systems, agencies and organisations to work together in order to improve the health outcomes of the communities they serve.’^30^

Community health is a multisector, multidisciplinary ‘collaborative enterprise that uses public health science, evidence-based strategies and other approaches to engage and work with communities, in a culturally appropriate manner, to optimise the health and quality of life of all persons who live, work or are otherwise active in a defined community or communities.’^33^ Fundamentally, population and community health practice applies public health concepts to populations defined by organisational or geo-cultural boundaries.

Prevention, through avoidance of risk factors or via prophylactic measures, occurs on primary, secondary and tertiary levels.^34^ Primary prevention intends to stop or reduce illness before it has occurred or developed. Secondary and tertiary prevention are focused on identifying disease early to intervene and reduce future harm or to maximise positive outcomes once a disease is already in progress. Primary prevention activities typically occur in context of community and family, often with little or no involvement from treatment-focused medical institutions.

Family physicians work at the interface between (1) the lives people live in their communities, which are impacted by social determinants, the environment and risk exposures; (2) the decisions people make about prevention and (3) the healthcare people receive when illness or injury occurs. Family physicians consider ever-widening levels of influence on health, including the family unit, the social support system, the community, the population, society overall, the environment, climate and much more.^35^ As inequities occur at each level, disparities in health outcomes emerge that must be corrected for health justice to be achieved.

**Figure 6:**
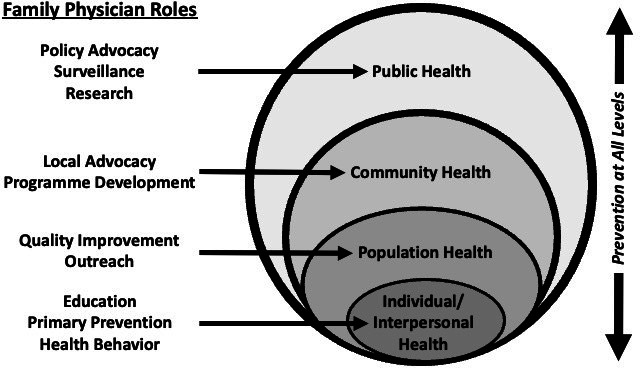
A socio-ecological model of public health.

This conceptual framework aligns well with a socioecological model of public health approaches to prevention. Family physicians can play important roles at multiple levels, such as being involved in advocacy related to policy decisions, collaborating with community organisations to improve local conditions, and treating patients and families (figure 6).^36^

Family physicians can think more broadly than the preventive guidelines for screening tests, vaccinations and lifestyle counselling. They can consider and act on the wider determinants of health. By considering the different levels within a socioecological framework of public health, family doctors can more effectively prevent illness and improve health.

### Readings

- American Academy of Family Physicians. Integration of primary care and public health (AAFP position paper). 2015, 2022. Available: https://www.aafp.org/about/policies/all/integration-primary-care.html [Accessed 31 January 2024].
- Campos-Outcalt D. Public health and family medicine: an opportunity. *J Am Board Fam Pract* 2004;17:207–11. doi: 10.3122/jabfm.17.3.207
- Sikora C, Johnson D. The family physician and the public health perspective: opportunities for improved health of family practice patient populations. *Can Fam Physician* 2009;55:1061–3.

## Information mastery in family medicine

Carina Brown and Dave Slawson

*When presented with clinical questions, family physicians apply the most valid and relevant evidence in the context of patients’ values and beliefs.*

The very foundations of evidence-based medicine rest with family physicians—considering patients’ preferences in the context of the clinical assessment and the best available evidence. One founder of evidence-based medicine wrote, ‘Good doctors use both individual clinical expertise and the best available external evidence.’^37^ With the markedly rapid growth of medical literature, family physicians must become information masters to successfully implement evidence-based medicine into clinical practice.^38^

Physicians-in-training quickly recognise that much of undergraduate medical education focuses on the ever-changing body of literature surrounding diagnostic tests, treatment and follow-up rather than common clinical questions. Medical schools train student physicians to memorise thousands of obscure facts, and much of this information relies on expert opinion—the lowest strength of a recommendation for an intervention or therapy. Medical education also commonly focuses on disease-oriented measurements, such as blood pressure, glomerular filtration rate, haemoglobin A1C and other surrogate markers of disease.

More often than not, disease-oriented outcomes do not accurately predict outcomes that patients or their physicians care about most. In the case of the treatment of hypertension, for example, students are taught that persistently elevated blood pressure damages arteries, leading to complications of stroke and cardiovascular events. For decades, the alpha-blocker doxazosin was used to lower blood pressure. From a mechanistic standpoint, this medication works: it lowers blood pressure by 5 mm Hg or more.^39^ Unfortunately, compared with other agents, doxazosin actually increases cardiovascular events. Just because a medication or intervention should improve outcomes, critical appraisal of the literature often finds the opposite is true.

Rather than focusing on the pathophysiology and mechanism of particular interventions, family medicine information masters who use evidence-based medicine study patient-oriented outcomes that matter.^40^ As well, they use ‘foraging’ tools to stay current with the best available evidence and ‘hunting’ tools to answer clinical questions at the point of care.^41^ Family physician must know where to look for the most valid and relevant current evidence to answer the clinical question at hand. With practice, information masters become skilled at finding the best available evidence with minimal effort.

**Figure 7:**
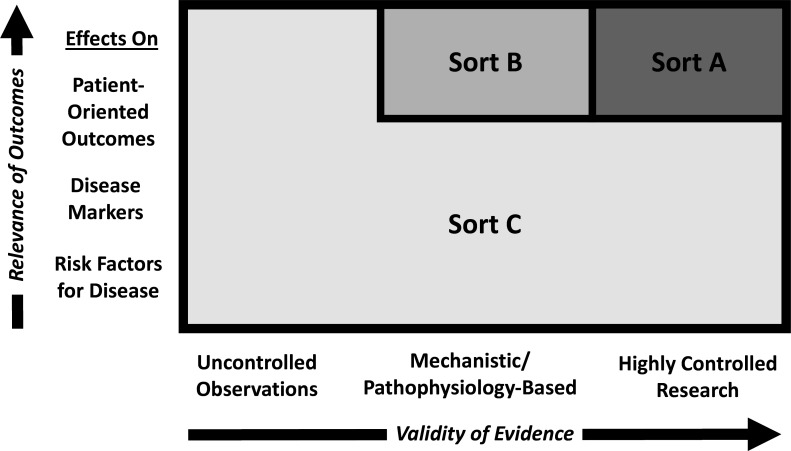
Making sense of clinical recommendations. Sort A=decision-making using high-quality, patient-oriented evidence; Sort B=decision-making using moderate-quality, inconsistent evidence; Sort C=traditional medical decision-making. Adapted with permission.^42^

Family physicians are inherently lifelong learners, acquiring new skills and new information to better care for their patients each day. As information masters, family physicians embody this philosophy using readily available tools to stay current as well as answer clinical questions (figure 7).^42^ Family physicians hold a unique place in the practice of medicine, where each day they meld clinical expertise, the most valid evidence and patient preferences to provide the best care for each patient.

### Readings

- Sackett DL, Rosenberg WMC, Gray J A M, Haynes RB, Richardson WS. Evidence based medicine: what it is and what it isn't. *BMJ* 1996;312:71–2. doi: 10.1136/bmj.312.7023.71
- Shaughnessy AF, Slawson DC, Becker L. Clinical jazz: harmonizing clinical experience and evidence-based medicine. *J Fam Pract* 1998;47:425–8.
- Slawson DC, Shaughnessy AF. Teaching evidence-based medicine: should we be teaching information management instead? *Acad Med* 2005;80:685–9. doi: 10.1097/00001888-200507000-00014

## Clinical courage

Jill Konkin, David Campbell, Ian Couper, Susan Williams, Robert Brooks and Lucie Walters

*Clinical courage is an umbrella term commonly used by rural physicians. It characterises how family physicians and other generalists make use of their knowledge, attitudes, skills, intentions and relationships for the benefit of patients in resource-limited environments.*

What is clinical courage? Clinical courage summarises the qualities that family physicians in rural areas possess that allow them to work at the edges of their usual scope of practice to care for patients. These qualities include the following^43^:

- Serving anybody and everybody in the community.
- Accepting uncertainty and persistently preparing for the unexpected.
- Deliberately understanding and marshalling resources in context.
- Humbly seeking to know one’s own limits.
- Clearing the cognitive hurdles when something needs to be done for patients.
- Providing collegial support to stand up again and again to address issues when needs demand.

**Figure 8:**
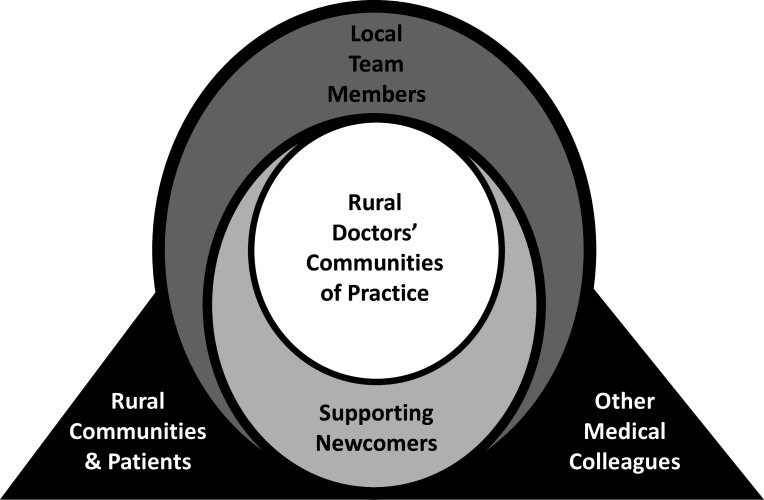
Relationships: the foundation of clinical courage. Adapted with permission.^44^

The relationships rural physicians have with local colleagues and patients, supportive specialist colleagues at a distance and the communities in which they live are key to developing and sustaining clinical courage in practice (figure 8).^44^

How do you develop clinical courage? For budding family physicians interested in developing clinical courage, choose medical education programmes that place one in rural communities for extended periods, preferably months on end. This will allow the time needed to build relationships and become valued members of the local healthcare team.

With the support of your teachers, you must, on a daily basis, intentionally acknowledge and work with uncertainty; you must also weigh the pros and cons of actions and inactions. Learn how your teachers work at the edge of their usual scope of practice to negotiate those spaces of uncertainty. Understand the resultant reflection these actions engender. Effective clinicians in rural practices understand what local resources are available and use these resources in innovative and creative ways in service of patients.

In this context, family physicians actively and regularly encounter circumstances in which they feel uncertain of their own capabilities. Not to worry! People learn best when faced with such uncertainty. Support from others, including colleagues, patients and community members, helps them navigate these uncomfortable spaces. These experiences help such family physicians develop the clinical courage needed to confidently work as rural generalist clinicians in resource-limited environments. Such experiences can help those choosing other careers in medicine to appreciate clinical courage as well.

Is clinical courage unique to rurally based family physicians? We encourage all learners in family medicine to develop and nurture the qualities of clinical courage. The best places to accomplish these tasks are in rural, remote and other under-resourced areas—these allow for increased hands-on experience and exposure to situations in which practitioners and learners regularly work at the edge of their competence and confidence.

Interested in developing clinical courage? To walk the path of clinical courage—to cultivate and nurture the above characteristics—we encourage you to seek out placements in rural settings, whether in medical school, residency training or clinical practice. We support judiciously exploring practice boundaries with your teachers while simultaneously balancing recognition of limits and clinical necessities. We also promote advocating for patients, families and communities challenged by the harsh realities of life in rural areas, remote from centres of wealth and power. Practising with clinical courage requires knowing oneself and one’s limits; you must know the resources available and have supportive relationships with colleagues, patients and community.

### Readings

- Konkin J, Grave L, Cockburn E, *et al*. Exploration of rural physicians’ lived experience of practising outside their usual scope of practice to provide access to essential medical care (clinical courage): an international phenomenological study. *BMJ Open* 2020;10:3 037 705. doi: 10.1136/bmjopen-2020–0 37 705
- Walters L, Couper I, Stewart RA, Campbell DG, Konkin J. The impact of interpersonal relationships on rural doctors’ clinical courage. *Rural Remote Health* 2021;21:6668. doi: 10.22605/RRH6668
- Couper I, Walters L, Williams S, *et al*. Exploring rural doctors’ experiences of coping with the emerging COVID-19 pandemic. *J Rural Health* 2022;38:923–31. doi: 10.1111/jrh.12654

## Data Availability

Data sharing not applicable as no datasets generated and/or analysed for this study.
